# Supplementary material for: Filamented Light (FLight) Bioprinting of Mini‐Muscles with Self‐Renewal Potential
Source: Adv Mater. 2025 Jul 23;37(40):e01147. doi: 10.1002/adma.202501147 (PMC12510276; doi:10.1002/adma.202501147)
Supplement: Supplementary file 1 — Supporting Information [file ADMA-37-e01147-s002.pdf]

ADVANCED MATERIALS

Supporting Information

for *Adv. Mater.*, DOI 10.1002/adma.202501147

Filamented Light (FLight) Bioprinting of Mini-Muscles with Self-Renewal Potential

*Hao Liu, Michael Winkelbauer, Jakub Janiak, Patrick Weber, Ali Kerem Kalkan, Inseon Kim, Parth Chansoria, Ori Bar-Nur and Marcy Zenobi-Wong**

Supplementary Materials for

Filamented Light (FLight) Bioprinting of Mini-Muscles with Self-Renewal Potential

Hao Liu *et al.*

*Corresponding author: Marcy Zenobi-Wong, marcy.zenobi@hest.ethz.ch

This PDF file includes:

Materials and Methods
Figures. S1 to S16
Tables S1 to S2

Other Supplementary Materials for this manuscript include the following:

Movies S1 to S6

Video S1: Spontaneous contraction of myotubes in FLight-engineered mini-muscles (pristine ColFib photoresin) after 2 weeks of differentiation.
Video S2: Spontaneous contraction of myotubes observed in bright-field (left) and calcium imaging (right; fluorescence intensity shown in color). Generated from time-lapse confocal microscopy scans.
Video S3: Myotube contraction in mini-muscles treated with different molecules, shown in bright-field (left) and calcium imaging (right; fluorescence intensity shown in color).
Video S4: Spontaneous contraction of myotubes in mini-muscles biofabricated using pristine ColFib photoresin, before and after CTX treatment.
Video S5: Spontaneous contraction of myotubes in mini-muscles created using GelNB photoresin, before and after CTX treatment.
Video S6: Spontaneous contraction of myotubes in mini-muscles (pristine ColFib photoresin) after 7 days of recovery from severe CTX-induced injury, treated with different molecules and concentrations.

Materials and Methods

Collagen-mimicking Peptide (GFOGER-3) and Fluorescent Labeling

GFOGER-3 peptide was purchased from BioCat GmbH (3-D Life GFOGER-3 Peptide, P12-1-CS). To prepare the gelatin-based photoresin containing GFOGER-3 peptide, GelNB was weighed and dissolved in 1× PBS as previously described. In parallel, a solution of GelSH and GFOGER-3 peptide (1 mg/mL in ultrapure water) was prepared such that the total amount of thiol (-SH) groups remained constant. Accordingly, the amount of GelSH was reduced to account for the thiol groups contributed by the GFOGER-3 peptide, ensuring an equivalent overall -SH concentration in the final resin formulation. For the synthesis of fluorescently labeled GFOGER-3 peptide, GFOGER-3 (0.3 mg, 80.4 µmol) was dissolved in 1× PBS at a concentration of 1 mg/mL. Fluorescein-5-maleimide (6.8 µg, 16.1 µmol; TCI Chemicals) was added from a 100× stock solution prepared in DMF. The reaction mixture was incubated at 4 °C overnight, followed by purification using a Hitachi Chromaster HPLC system (Hitachi) equipped with a Poroshell 120 CS-C18 column (Agilent Technologies). Product-containing fractions were pooled and lyophilized. The final yield was 18.9 mg (50.7 µmol), corresponding to a 63% recovery. The degree of substitution (~3.6%) was determined by dissolving the product in deionized water and measuring absorbance on a NanoDrop One^C spectrophotometer (Thermo Fisher Scientific).

Integrin Blocking

An integrin beta 1 function-blocking antibody (AIIB2) was obtained from the Developmental Studies Hybridoma Bank (DSHB). AIIB2 is a rat monoclonal IgG1 antibody that specifically binds to the extracellular domain of integrin beta 1, thereby downregulating integrin beta 1–mediated signaling. The AIIB2 stock solution (162 µg/mL) was diluted in culture medium to a final concentration of 5 µg/mL. As a control, rat IgG (Thermo Fisher Scientific) was added to the culture medium at the same final concentration of 5 µg/mL. Antibody-containing medium was replaced every 2 days throughout the culture period.

Benchmarking of Different Biofabrication Approaches

Photoresins (ColFib) used in different biofabrication approaches were prepared in the same manner. To visualize the ColFib matrix, collagen and fibrinogen were fluorescently labeled with FITC or Rhodamine. Briefly, lyophilized pristine proteins were dissolved in 0.1 M carbonate buffer (pH = 9.0), and either 5/6-carboxyfluorescein succinimidyl ester (NHS-FITC) or 5/6-carboxy-tetramethyl-rhodamine succinimidyl ester (NHS-Rhodamine)was added at a final concentration of 0.1 mg/mL. The conjugation reaction was carried out overnight at 4 °C in the dark. The product was purified by dialysis against PBS at 4 °C for fibrinogen and against acidified milliQ water for collagen, protected from light, and subsequently lyophilized.
For post-seeding of cells onto FLight hydrogels, acellular printed hydrogel sheets were washed in 1× PBS for 30 minutes. Cells were then seeded onto the surface at a density of 0.2 million cells/cm^2. For hydrogel casting, a mixture of cells and photoresin was dispensed into a PDMS mold and photocrosslinked under a UV box. The same light dose was applied across all samples to ensure comparable stiffness. Once crosslinked, the hydrogel constructs were manually transferred into a two-pin anchoring system using metal needles. For volumetric 3D printing, a hydrogel sheet design (identical in dimensions to the FLight-printed mini-muscle construct) was uploaded to a commercial volumetric printer (Tomolite, Readily3D SA). The same light dose was used to print cellular constructs. All engineered tissue samples were cultured in

growth medium (GM) for 7 days, followed by differentiation medium (DM) for an additional 14 days.

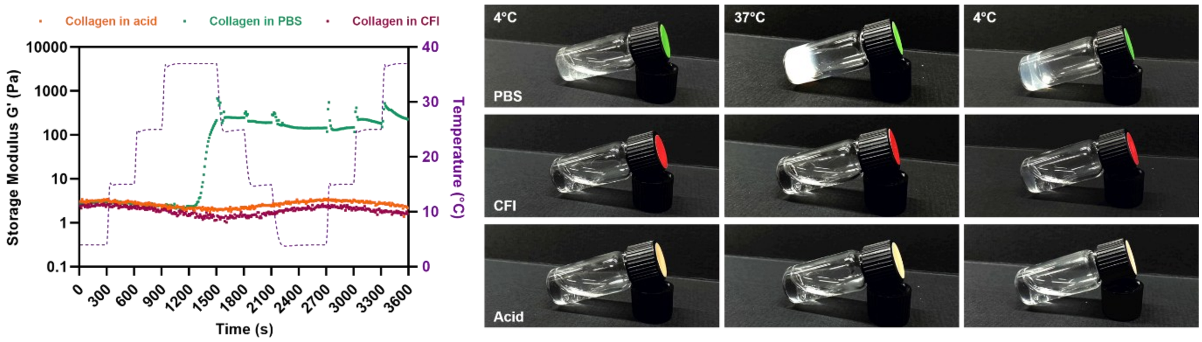

Figure S1. Thermo-crosslinking of collagen in different solutions. Rheological analysis of thermo-crosslinking of 5 mg/mL collagen in PBS solution (pH≈7.4), collagen fibrillogenesis inhibition (CFI) solution (pH≈7.2), and in acetic acid (pH≈3.1). Irreversible thermal crosslinking was confirmed in PBS solution but was in CFI or under acidic conditions.

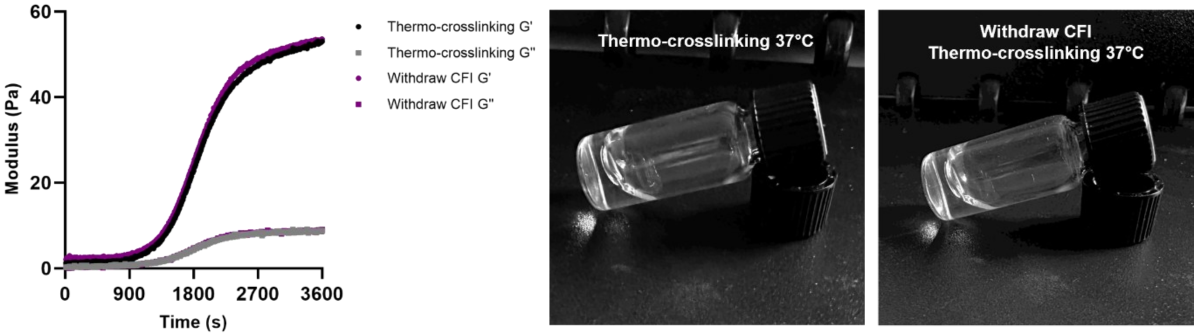

Figure S2. Thermo-crosslinking of collagen and after removal of CFI solution. Rheological analysis of thermo-crosslinking of 3 mg/mL collagen in PBS solution (pH≈7.4) and after removal of CFI by dialysis (40 kDa) and redissolved in PBS solution (pH≈7.4). The similar thermo-crosslinking behavior indicates that the inhibition of collagen self-assembly by CFI is transient. The collagen crosslinking at 37°C was still present after removal of CFI solution.

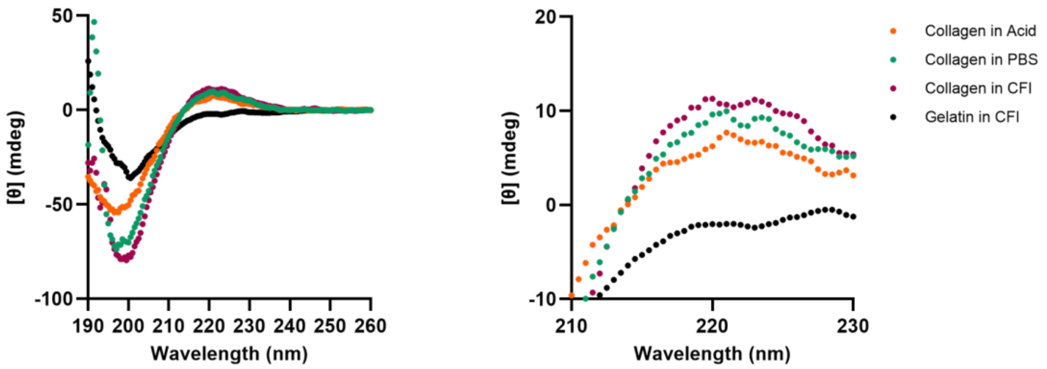

Figure S3. Circular dichroism (CD) spectra of collagen type I (0.1 mg/mL) in different solutions, with gelatin in CFI at the same concentration included as a control.

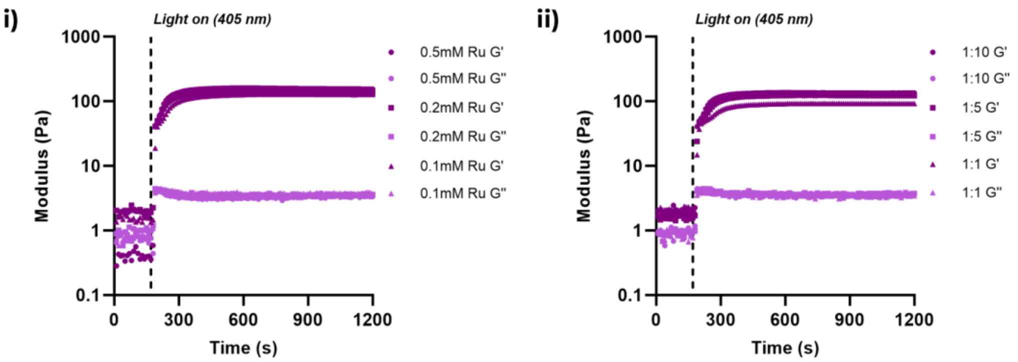

Figure S4. Photorheological studies of collagen with varying concentrations of photoinitiator. Rheological analysis of thermo-crosslinking of 3 mg/mL collagen in CFI solution with different concentrations of Ru and the ratio of Ru to SPS. **i**) with different Ru concentrations but a fixed ratio of Ru to SPS (1:10) and **ii**) with different Ru-SPS ratios but a Ru concentration of 0.2 mM.

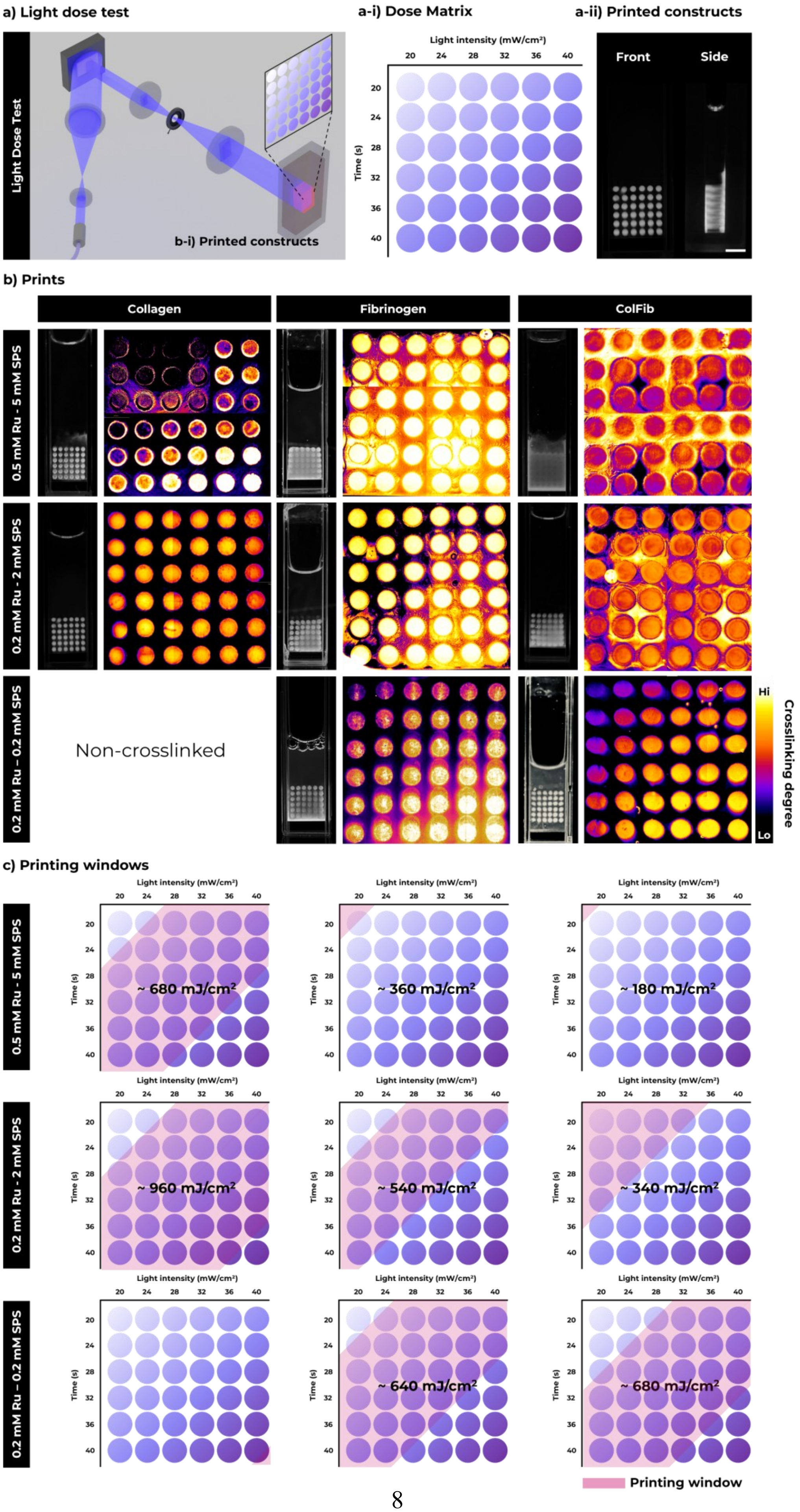

a) Light dose test
a-i) Dose Matrix
a-ii) Printed constructs
Light Dose Test
b-i) Printed constructs
Light intensity (mW/cm²)
Time (s)
Front
Side
b) Prints
Collagen
Fibrinogen
ColFib
0.5 mM Ru - 5 mM SPS
0.2 mM Ru - 2 mM SPS
0.2 mM Ru – 0.2 mM SPS
Non-crosslinked
Hi
Lo
Crosslinking degree
c) Printing windows
~ 680 mJ/cm²
~ 360 mJ/cm²
~ 180 mJ/cm²
~ 960 mJ/cm²
~ 540 mJ/cm²
~ 340 mJ/cm²
~ 640 mJ/cm²
~ 680 mJ/cm²
Printing window

Figure S5. Light dose test of FLight biofabrication using different photoresin formulations. a) Schematic of the light dose test designed to evaluate the printability and optimal crosslinking window of various photoresins. A 6 × 6 array of cylindrical patterns (1 mm diameter, 0.5 mm spacing) with different light doses was projected onto a cuvette filled with photoresin as previously reported.[1] **b)** Photography and fluorescence images of printed cylinder arrays, showing differences in crosslinking outcomes across formulations. Tested photoresins include: Collagen (5 mg/mL), Fibrinogen (50 mg/mL), and a ColFib mixture (Collagen 3 mg/mL + Fibrinogen 25 mg/mL). **c)** Light dose matrix and corresponding printing window (depicted in light pink). An optimal dose is defined as the condition in which a hydrogel cylinder is fully crosslinked (i.e., measured diameter corresponds to 1 mm) without fusing to adjacent constructs (i.e., overcrosslinking).

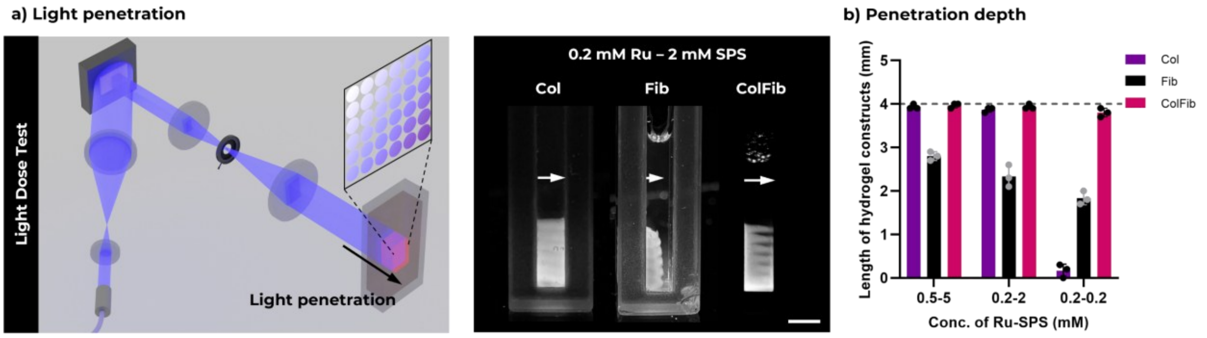

Figure S6. Light penetration depth and construct length in FLight-printed hydrogels using different photoresin formulations. a) Schematic and side-view images of printed hydrogel cylinders generated via light dose testing. Constructs were printed in a cuvette to evaluate horizontal light penetration (i.e., length along the light projection axis) across formulations. Scale bar: 5 mm. **b)** Analysis of construct length along the direction of light projection, representing the effective light penetration depth within each photoresin formulation. The dashed line indicates the inner length of the cuvette (4 mm), serving as a reference for an optimized photoresin that achieves complete crosslinking throughout the entire construct. Photoresins evaluated in this study include pure collagen (5 mg/mL), pure fibrinogen (50 mg/mL), and a composite ColFib formulation (collagen 3 mg/mL + fibrinogen 25 mg/mL).

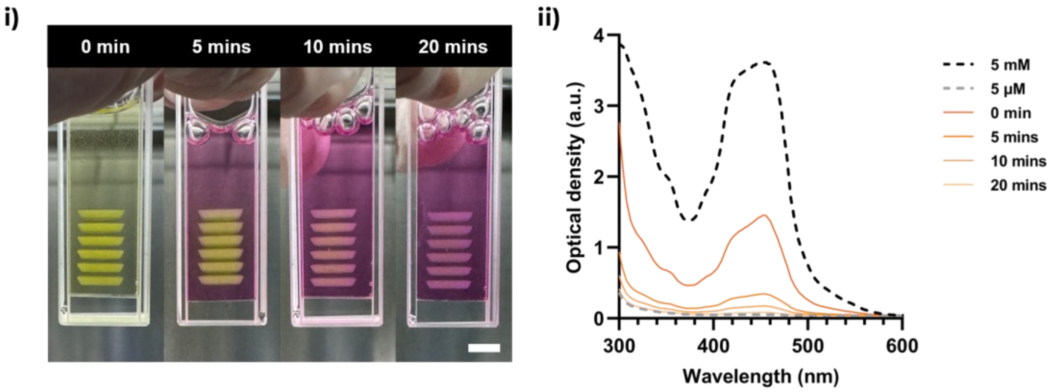

Figure S7. Removal of ruthenium (Ru) after FLight biofabrication. i) Photograph of printed FLight muscle constructs with Ru-SPS after 0, 5, 10, and 20 min of washing. Scale bar: 5 mm. **ii**) Optical density of photoabsorption with a wavelength range of 300-600 nm. A reduced absorption peak near 460 nm after 5, 10, and 20 min of washing indicates the decrease of Ru concentration in printed hydrogel constructs. After 20 min of washing, the absorbance approaches that of a standard Ru solution with a concentration of 5 µM.

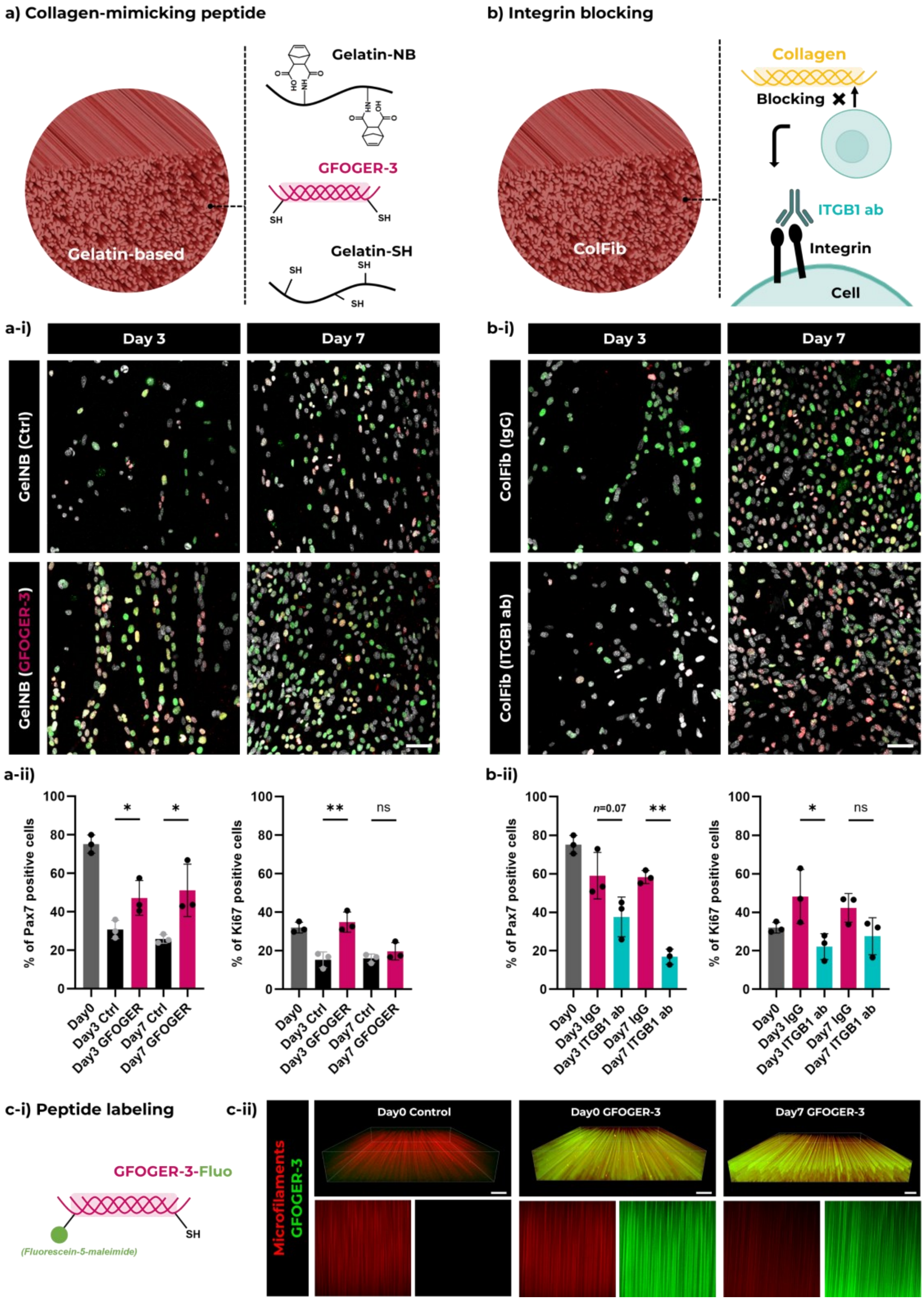

Figure S8. Investigation of the effect of cell–matrix interactions on the cellular state of Pax7-nGFP myoblasts. a) Schematic of enhanced cell–matrix interactions via functionalization of the GelNB-GelSH matrix with collagen-mimicking peptides (GFOGER-3), which form a triple-helix structure. **a-i)** Representative confocal images (maximum intensity projections) of encapsulated Pax7-nGFP myoblasts in peptide-modified FLight hydrogels. **a-ii)** Quantitative analysis of $Pax7^{+}$ and $Ki67^{+}$ cell populations. Scale bar: 50 µm. **b)** Schematic of reduced cell–matrix interactions through integrin beta 1 blockade using a blocking antibody

(ITGB1 ab). **b-i)** Representative confocal images of mini-muscle constructs cultured in medium containing either integrin beta 1 antibody or IgG control. Scale bar: 50 µm. **b-ii)** Quantification of $Pax7^+$ and $Ki67^+$ cell populations under both conditions (n=3). **c)** Imaging-based validation of collagen-mimetic peptide immobilization. The SH group was partially substituted with fluorescein-5-maleimide for visualization. Confocal images confirm successful peptide incorporation and retention in the hydrogel matrix for up to 7 days. Scale bars: 50 µm.

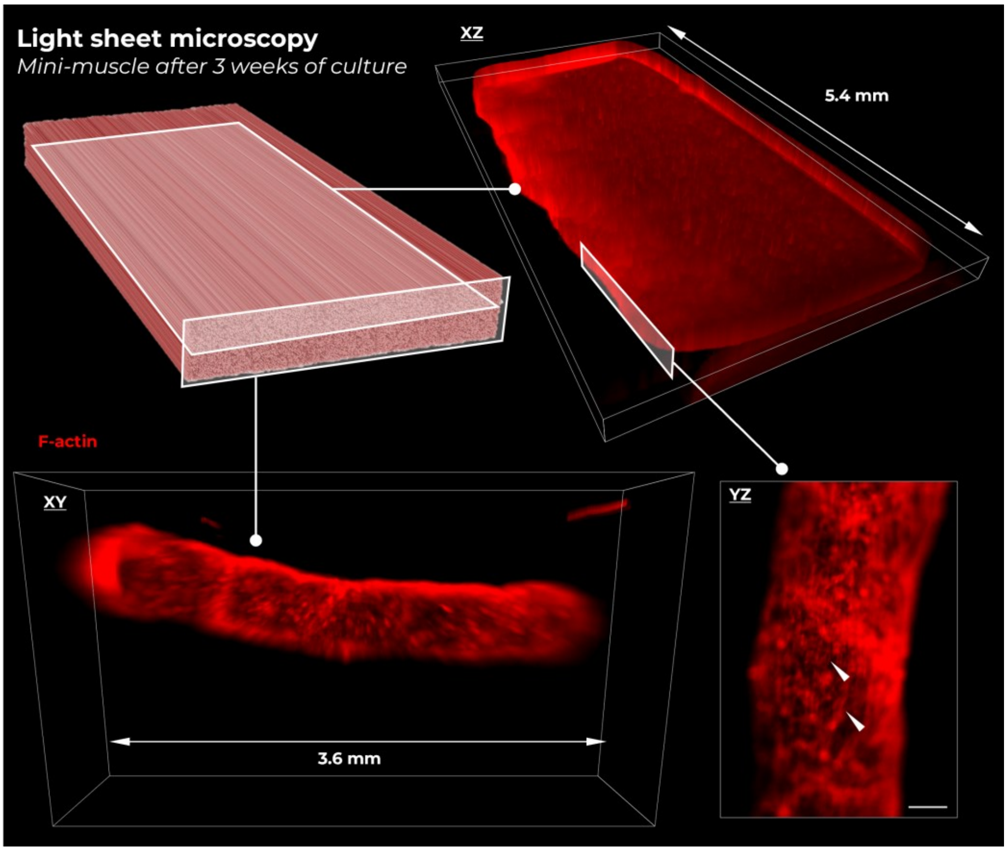

Figure S9. Light sheet microscopy images of mini-muscle constructs after 3 weeks of culture. Cross-sectional views (XY, XZ, and YZ) were reconstructed or captured from 3D volumetric scans acquired using an axially scanned light sheet microscope (MesoSPIM V4) equipped with a Mitutoyo M Plan Apo 5×/0.14 objective and a 561 nm laser. White arrows in the YZ panel indicate aligned F-actin. Scale bar (YZ panel): 200 µm.

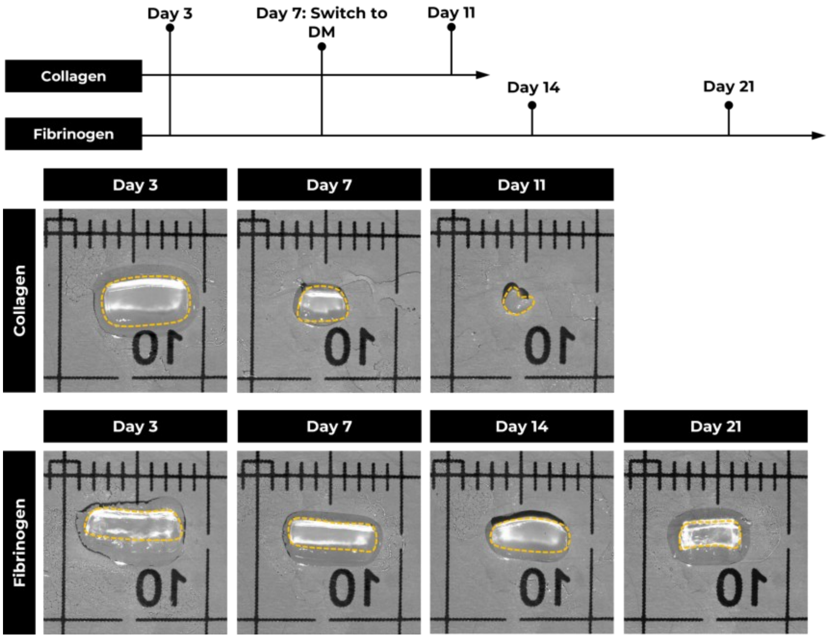

Figure S10. Timeline and representative images of mini-muscle constructs printed using either collagen or fibrinogen-based photoresins. Collagen-based constructs exhibited significant shrinkage and poor structural integrity by day 11 of culture. In contrast, fibrinogen-based constructs maintained better shape fidelity and remained stable up to day 21. Yellow dashed lines outline the shape and dimensions of the constructs at each time point. Photoresin formulations: Collagen (5 mg/mL); Fibrinogen (50 mg/mL).

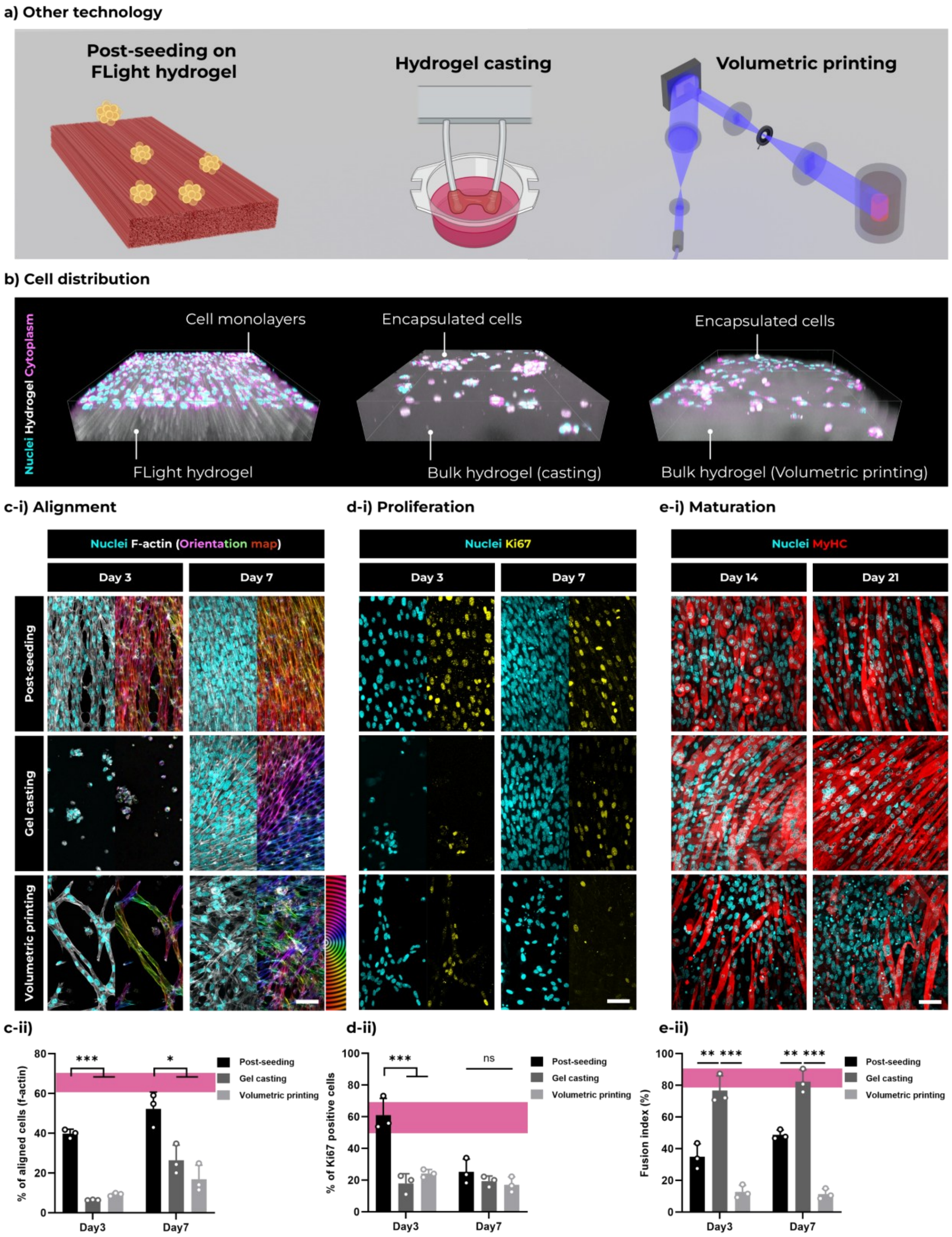

Figure S11. a) Schematic illustration of engineered muscle constructs fabricated using different biofabrication strategies. All constructs were produced using the same ColFib photoresin formulation and identical light doses. **b)** Representative 3D views of cells and hydrogel matrix obtained from confocal z-stack scans. Cells were either post-seeded on the hydrogel surface or encapsulated during the fabrication process. Images depict cell distribution and morphology on day 3. **c–e)** Representative confocal images and quantitative analyses of cell alignment, proliferation, and myogenic maturation at various time points across different biofabrication methods (n=3). Scale bars: 50 µm. The pink shaded areas highlight the performance metrics of

FLight-printed mini-muscles for each corresponding evaluation. Results are presented as mean ± SEM; *ns*: not significant, $*P < 0.05$, $**P < 0.01$, and $***P < 0.001$.

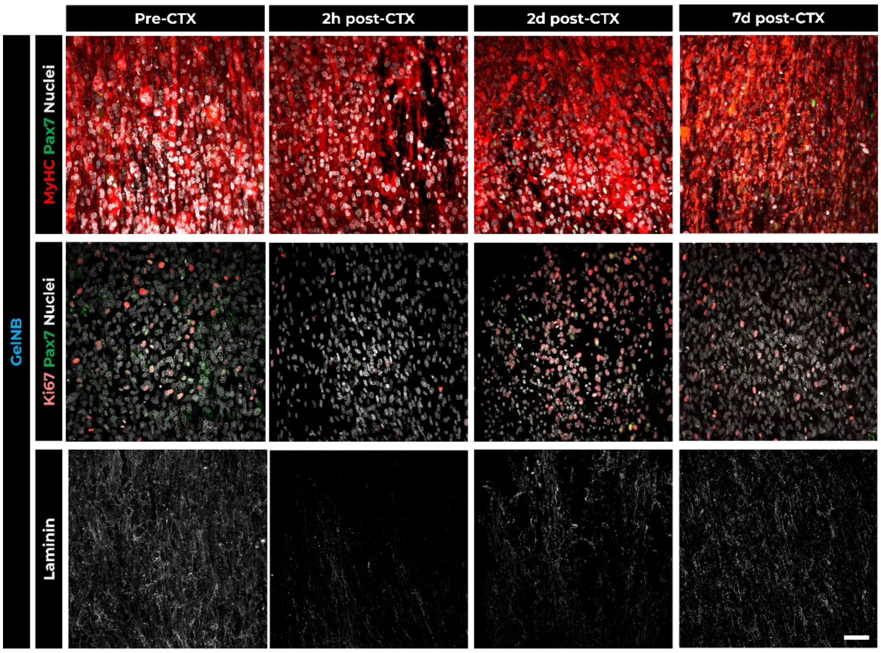

Figure S12. Self-renewal potential of engineered FLight muscle biofabricated using GelNB photoresin. Representative confocal images of engineered FLight muscle after 21 days of culture (Pre-CTX), and 2 hours (2h post-CTX), 2 days (2d post-CTX) and 7 days (7d post-CTX) after CTX treatment. Scale bar: 50 µm.

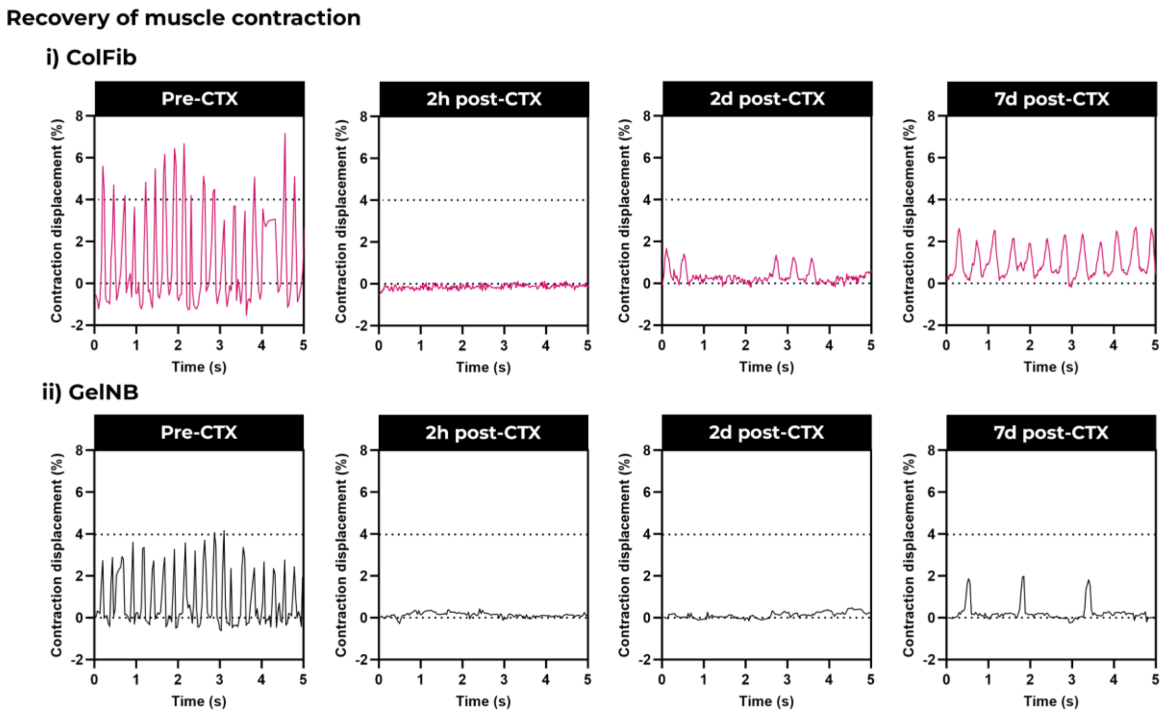

Figure S13. Spontaneous contraction analysis of engineered FLight muscles biofabricated from **i**) ColFib and **ii**) GelNB at 2h, 2 days and 7 days after CTX treatment.

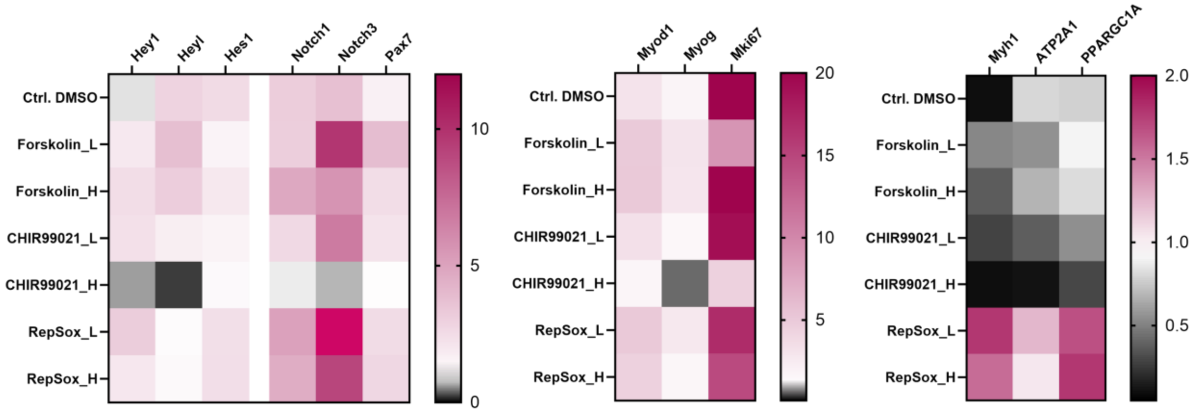

Figure S14. Gene expression profile of mini-muscles treated with different molecules and concentrations. Relative change of gene expression in mini-muscles after 7 days of recovery. The fold change shown in the heat map is the mean value. All data were collected from three biological replicates of hydrogel samples with two technical replicates per tissue sample. Details of gene expression are depicted in Figure S15.

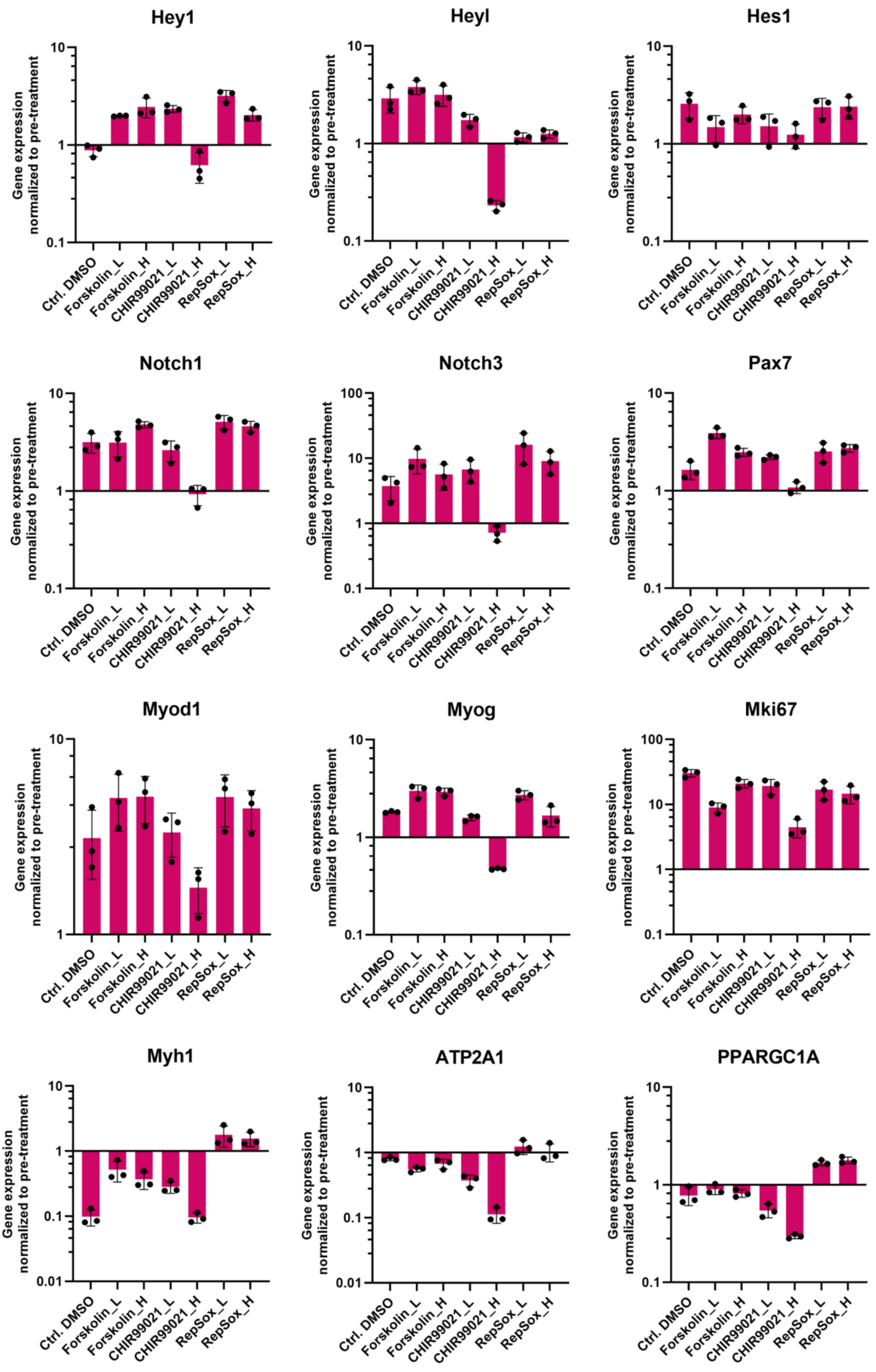

Figure S15. Extend gene expression profile of mini-muscles treated with a range of small molecules and doses.

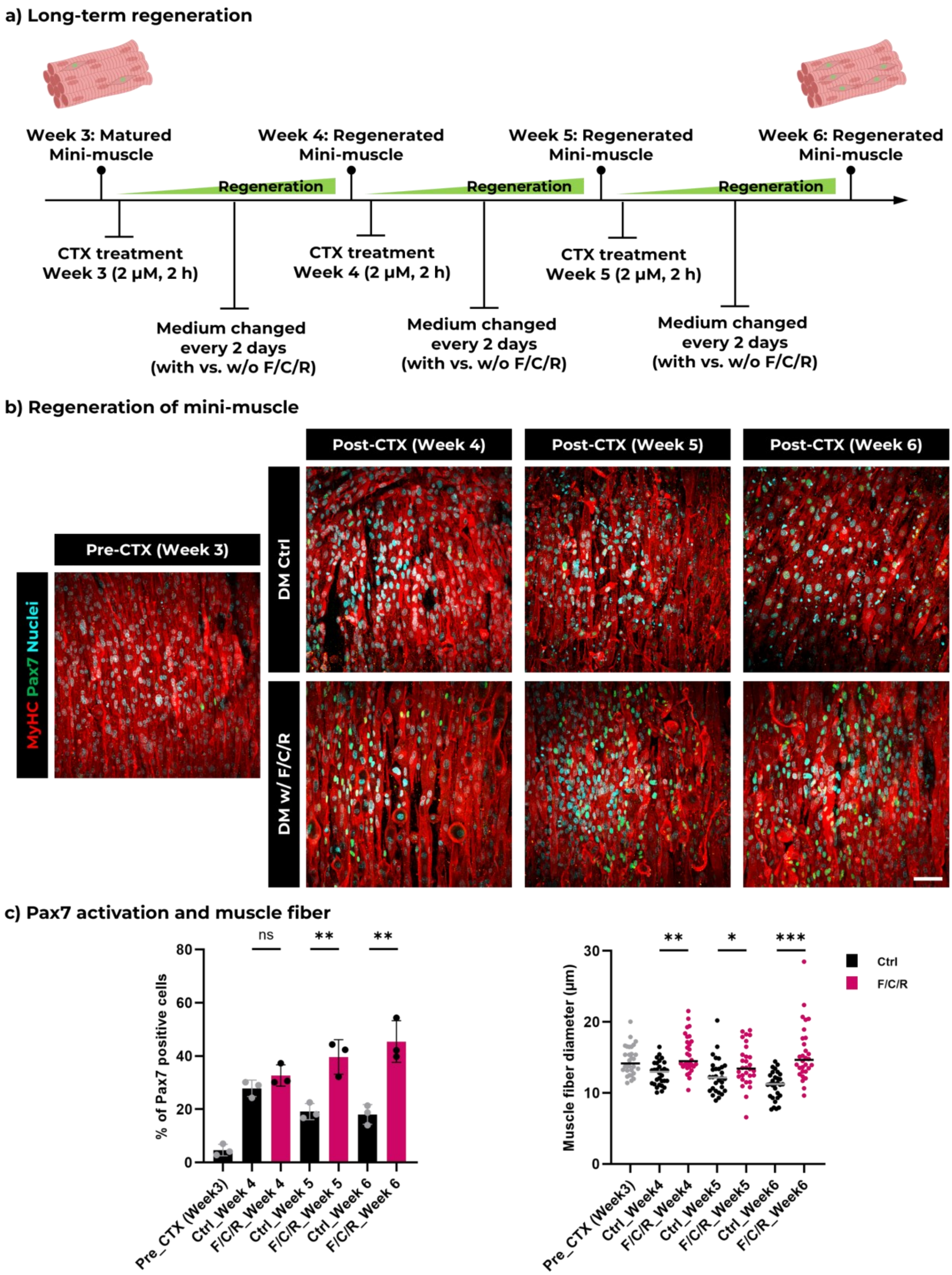

Figure S16. Schematic and timeline of long-term regeneration studies in mini-muscle constructs. a) After 3 weeks of maturation, mini-muscles were subjected to three rounds of CTX-induced injury (one treatment every 7 days). Constructs were then cultured either in

standard differentiation medium (DM, control) or DM supplemented with Forskolin (F), CHIR99021 (C), and RepSox (R) (F/C/R; 2 µM/2 µM/2 µM), with medium changed every 2 days. **b**) Representative confocal images of mini-muscles at each time point, generated from 3D scans using maximum intensity projection. Scale bar: 50 µm. **c**) Quantitative analysis of $Pax7^{+}$ cell populations and muscle fiber diameters under different treatment conditions. Data were measured from 3D confocal scans as depicted in panel b) ($n \geq 3$). Results are presented as mean ± SEM; *ns*: not significant, $^{*}P < 0.05$, $^{**}P < 0.01$, and $^{***}P < 0.001$.

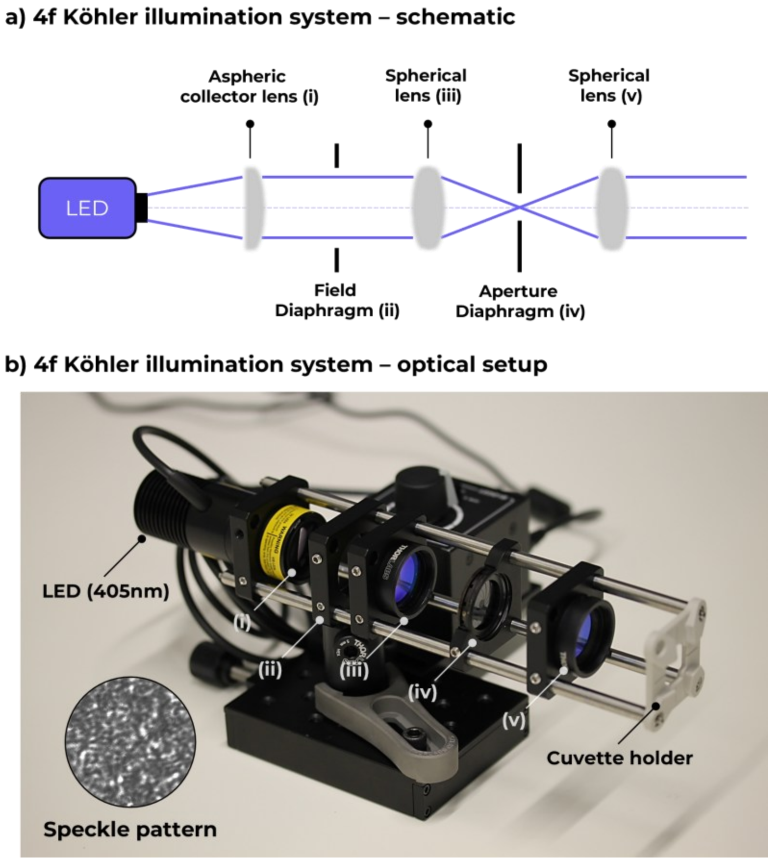

Figure S17. a) Schematic illustration of a 4f Köhler illumination system, in which a 405 nm LED source can produce a spatially coherent light beam capable of inducing hydrogel microfilament formation in non-linear media.[2,3] **b**) Photograph of a compact, miniaturized optical device assembled with the 4f Köhler illumination configuration.

Table S1: Antibodies and fluorescent dyes used in this study.

Resources	Manufacturer	Catalog	Host Species	Dilution
**Primary antibodies**				
Anti-Ki-67	BD Biosciences	550609	Mouse	1:200
Anti-MyoD	Invitrogen	PA5-23078	Rabbit	1:100
Anti-ITGB1	Invitrogen	14-0299-82	Mouse	1:100
Anti-MyHC	DSHB	MF20	Mouse	1:10
Anti-Sarcomeric alpha actinin	Sigma	A7732	Mouse	1:200
Anti-Laminin	Invitrogen	PA1-16730	Rabbit	1:200
**Secondary antibodies & Dyes**				
Hoechst 33342	Invitrogen	H3570	-	1:1000
Phalloidin-Tetramethylrhodamin B	Sigma	P1951	-	1:1000
Goat anti-Mouse IgG(H+L), Alexa 568	Invitrogen	A-11004	-	1:500
Goat anti-Rabbit IgG(H+L), Alexa 647	Invitrogen	A-21244	-	1:500

**All antibodies were diluted in 1% BSA-PBS solution if not indicated.*

Table S2: Sequence of the primer pairs used in the qPCR studies.

Gene	Reference	Forward Primer	Reverse Primer
GAPDH	NM_008084.4	AAGGTCGGTGTGAACGGATTT	GAGGTCAATGAAGGGGTCGT
Hes1	NM_008235.3	GGTGCTGATAACAGCGGAAT	TTAGGGCTACTTAGTGATCGGT
Hey1	NM_010423.2	GCCTTTGAGAAGCAGGGATCT	CGTGCGCGTCAAAATAACCT
Heyl	NM_013905.3	AAGAAGCGCAGAGGGATCATA	TTTCTCAAAGGCAGTGGGGAC
Pax7	NM_011039.3	TCTTACTGCCCACCCACCTA	CCAGGTAATCAACAGCAGTTTGG
Notch1	NM_008714.3	TGGGCTCCTAACACCTGACT	TGGGGATCAGAGGCCACATA
Notch3	NM_008716.3	CTCCAGATGCCTGTGAGTCC	CACACTGATGGCCCTGGAAT
Mki67	NM_001081117.2	ACCCTAGAGGATCTGCCTGG	TCGGGCATCTTTGGGGTTTT
Myod1	NM_010866.2	GCTCTGATGGCATGATGGATT	ACTGTAGTAGGCGGTGTCGTA
Myog	NM_031189.2	GTGAATGCAACTCCCACAGC	CGCGAGCAAATGATCTCCTG
Myh1	NM_030679.2	TCCTCATAAAGCTTCAAGTTTGGAC	TATTGGTTGCAGCCCAGTGA
ATP2A1	NM_007504.2	AAGGCGAAGAAACCGTCACT	GCGTTCTCTGCATTCCGTTC
PPARGC1A	NM_008904.3	GCTGTGTGTCAGAGTGGATTG	AGCAGCACACTCTATGTCACT

Supplementary References

[1] R. Rizzo, D. Ruetsche, H. Liu, M. Zenobi-Wong, *Advanced Materials* **2021**, *33*, 2102900.

[2] J. Madrid-Wolff, M. F.-S.-T. Biophysicist, undefined 2020, *meridian.allenpress.comJ Madrid-Wolff, M Forero-SheltonThe Biophysicist, 2020•meridian.allenpress.com* **n.d.**

[3] H. Liu, P. Chansoria, P. Delrot, E. Angelidakis, R. Rizzo, D. Rütsche, L. A. Applegate, D. Loterie, M. Zenobi-Wong, *Advanced Materials* **2022**, *34*, 2204301.
